# Synthesis, radiolabeling, and evaluation of a ^68^Ga-labeled tyrosine kinase inhibitor for detecting EGFR^T790M^ mutations *in vivo*

**DOI:** 10.3389/fbioe.2026.1741512

**Published:** 2026-01-21

**Authors:** Jiajun Xie, Weiguo Xu, Hui Deng, Xiaoai Wu, Jing Zhu

**Affiliations:** 1 NHC Key Laboratory of Nuclear Technology Medical Transformation (MIANYANG CENTRAL HOSPITAL), Mianyang, China; 2 Precision Medicine Key Laboratory of Sichuan Province, West China Hospital of Sichuan University, Chengdu, China; 3 Department of Respiratory and Critical Care Medicine, Mianyang Central Hospital, School of Medicine, University of Electronic Science and Technology of China, Mianyang, China; 4 Department of Nuclear Medicine, West China Hospital, Sichuan University, Chengdu, China; 5 Sichuan Provincial Engineering Research Center of Radiopharmaceutical Clinical Translation, Sichuan University, Chengdu, Sichuan, China

**Keywords:** EGFR, lung cancer, PET, radiotracer, T790M

## Abstract

Radiolabeled tyrosine kinase inhibitors (TKIs) have been used in clinics for the detection of epidermal growth factor receptor (EGFR) mutations in non-small-cell lung cancer (NSCLC) patients by positron emission tomography (PET), and the potential responder for TKI targeted therapy can be selected and benefited in subsequent TKI-based anti-tumor treatment. However, as activated T790M resistance mutation is often observed in NSCLC patients underwent EGFR TKI based therapies, the detection of EGFR mutation status is of great importance to evaluate therapeutic efficacy and prolongs overall survival with these patients. In addition, no radiotracer has been reported with the ability to detect EGFR^T790M^ mutation *in vivo* at present. Based on the chemical structure of Rociletinib, a novel radiolabeled PET tracer (^68^Ga-**1**) was developed with potent binding affinity *in vitro (*with IC_50_ value of 9.21 nM*)*. In addition, ^68^Ga-**1** also showed promising properties in detection EGFR^T790M^ mutation in the subsequent *in vitro* and *in vivo* evaluations. Herein we report the synthesis, radiolabeling, and preclinical evaluation of ^68^Ga-**1** for detecting EGFR^T790M^ mutation, and our result indicated that Rociletinib may be regarded as a lead compound to develop a selective EGFR^T790M^ PET tracer with further modification and optimizations.

## Introduction

**FIGURE 1:**
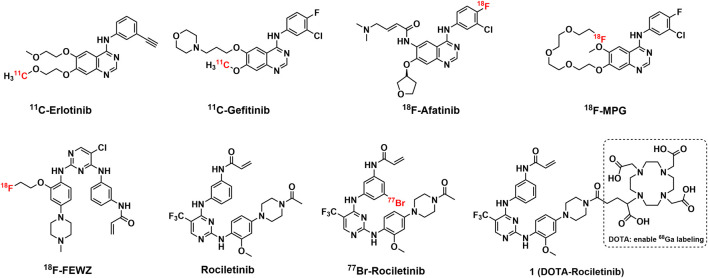
Structures of representative reported radiotracers based on first-generation EGFR TKIs and Rociletinib with its analogues.

It is considered that the EGFR mutation is the driving force in NSCLC patients since a great percentage of patients can be detected with EGFR gene alteration and overexpression (Sharma et al., 2007; Sequist et al., 2015; Zheng et al., 2016). As a subfamily member of receptor tyrosine kinase, EGFR plays crucial roles in signaling pathways related to cellular survival, multiplication, differentiation and metastasis (Zheng et al., 2016; Bahce et al., 2017). Activating EGFR was thus regarded as an attractive biomarker for tumor imaging and drug development in recent years (Bernard-Gauthier et al., 2015; Bahce et al., 2017). During the last decades, EGFR TKIs have demonstrated their potential to improve clinical outcomes in targeted therapies, with an overall response rate of about 80% in NSCLC patients harboring activating EGFR alterations (Yeh et al., 2011; Sun et al., 2018). The EGFR mutation status is of great importance to predict the clinical outcome of TKI based therapy. Therefore, multiple technical platforms have been developed for genotyping EGFR, including non-invasive PET imaging (Yeh et al., 2011; Bernard-Gauthier et al., 2015; Bahce et al., 2017; Sun et al., 2018). Based on highly specific radiotracers, non-invasive PET/CT images have been used to detect the sensitive EGFR mutation status before and/or after TKI treatment, which showed superior properties compared with other techniques. Over 30 TKI based PET tracers have been reported with the ability to detect EGFR sensitive mutations at present, and several tracers have been evaluated in clinical (see Figure 1) (Bernard-Gauthier et al., 2015; Bahce et al., 2017; Zhu et al., 2022).

Although favorable clinical outcome can be achieved with first-and second-generation EGFR TKI based therapies, a majority of patients (up to 70%) experience disease progression due to acquired resistance following a period of 9–15 months of EGFR TKI treatment (Wu et al., 2023). The predominant mechanism behind TKI resistance is a secondary EGFR alteration known as T790M, located at the gatekeeper site (position 790) within exon 20. This specific mutation accounts for nearly 60% of acquired resistance instances in NSCLC cases (Yun et al., 2008; Husain et al., 2017; Leonetti et al., 2019). Therefore, the detection and monitoring of EGFR^T790M^ mutation status during EGFR-TKI treatment is essential to maintain and predict therapeutic efficacy (Ward et al., 2013; Yu et al., 2013; Finlay et al., 2014). Nevertheless, only countable radiotracers that targeted EGFR^T790M^ have been developed and reported to date (Goggi et al., 2019; Fawwaz et al., 2021) (see Figure 1).

Derived from highly potent inhibitor (WZ002) of EGFR^T790M^, ^18^F-FEWZ displayed limited specificity and low binding to the target due to the rapid blood clearance. Although radiobrominated Rociletinib displayed specificity in cell uptake studies, the low *in vivo* tumor accumulation in biodistribution studies indicated the tracer was insufficient for tumor visualization with PET. In addition, no *in vivo* PET studies were performed with EGFR^T790M^ targeted PET tracers at present.

Held same “Aniline-Pyrimidine-Aniline” scaffold, current EGFR^T790M^ targeted PET tracers with higher lipophilicity displayed limited tumor accumulation, we believe the introduction of more hydrophilicity moiety to the molecule would enhance the ability to cross/pass through multiple membranes for tumor engagement and retention.

To develop potent PET tracers that selectively target EGFR^T790M^ for invasive detection and visualization, Rociletinib attracted our attention. Rociletinib showed potent and selective activity against EGFR^T790M^ in both pre-clinical and clinical studies (Walter et al., 2013; Sequist et al., 2015). Although Rociletinib has not been approved by FDA for multiple reasons, the pyrimidine-based scaffold can be regarded as an ideal “warhead” with high affinity for developing EGFR^T790M^ targeted PET tracers. Bearing several hydrophilicity groups such as carboxylic acid and amine, current chelators such as DOTA(1,4,7,10-tetraazacyclododecane-1,4,7,10-tetraacetic acid), and NOTA(2,2′,2''- (1,4,7-triazonane-1,4,7-triyl)triacetic Acid) display higher hydrophilicity, and the introduction of these chelators would improve the pharmacokinetics properties of desired molecules. In addition, the introduction of chelators moiety enables the therapeutic radionuclide labeling (such as ^177^Lu and ^161^Tb) to the potent “warhead” for the development of Targeted-Radionuclide-Therapy pharmaceuticals. In this investigation, a potent ^68^Ga radiolabeled Rociletinib derivative (^68^Ga-**1**) was successfully designed and prepared with the introduction of DOTA moiety (for radiometal labeling) and evaluated *in vitro*, and further *in vivo* evaluations for detecting EFGR^T790M^ were also performed and discussed.

## Materials and methods

### Molecular docking studies

To evaluate the binding model of the designed compound with EGFR^T790M^, molecular docking was performed. All calculations were performed on Discovery Studio (Accelrys Inc., Version 3.1, United States). The protein structure of EGFR^T790M^ was prepared with a previously published crystal structure (PDB ID: 2JIT) (Yan et al., 2017). The binding of the compound with the target protein was allocated as a sphere from its original reported ligand, and the space size is large enough to incorporate the binding site of EGFR^T790M^ with the ligand. All docking calculations were performed on GOLD program.

### General information for chemical synthesis

All regents, solvents and consumables used in this study were obtained from commercial sources and used without further processing unless indicated. Thin-layer chromatography (TLC) with silica gel plates for monitoring the organic reactions, and flash column chromatography was used for the separation and purification of crude products. ^1^H and ^13^C NMR tests were performed on an AV-400 Bruker spectrometer in the Analysis and testing center of Sichuan university. Mass spectra were obtained and recorded on an Agilent 6125 LC/MSD system. Compound **1** was synthesized following an identical procedure for Rociletinib, with minor modifications (Walter et al., 2013; Ward et al., 2013; Fawwaz et al., 2021).

**SCHEME 1:**
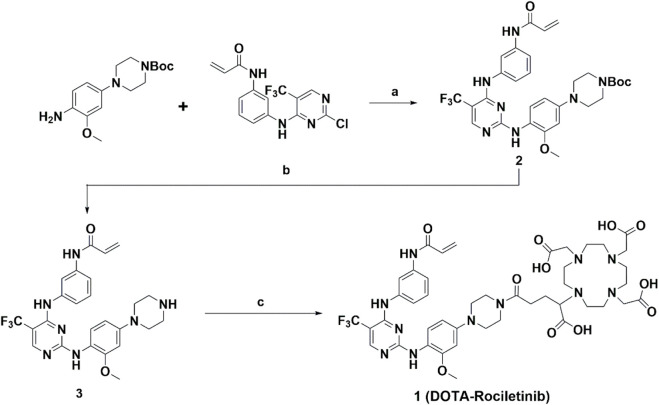
Synthesis of DOTA-Rociletinib (compound **1**). Conditions: (a) Trifluoroacetic Acid, 2-Butanol, overnight; (b) Trifluoroacetic Acid, dichloromethane, r. t 4h; (c) Triethylamine, DOTAGA-anhydride, dimethyl sulfoxide, overnight.

The synthesis route is outlined in Scheme 1 and all detailed information about the synthesis procedures was provided in the supporting materials, as well as the characterization data.

### Biochemical activity test

EGFR Kinase inhibition tests (Lantha screen Assay) for compound **1** were performed by ChemPartner Co., Ltd. (Shanghai, China) with Staurosproine and Rociletinib as positive control.

### ^68^Ga radiolabeling

^68^GaCl_3_ was eluted from a^68^Ge/^68^Ga generator (Eckert & Ziegler, Germany) by 5 mL of 0.1 N HCl solution and used for radiolabeling according to a classic DOTA based radiolabeling procedure published previously (Lindner et al., 2018; Giesel et al., 2019). Briefly, the generator solution containing ^68^GaCl_3_ (400 μL) was added into the reaction mixture of 40 μL of compound **1** and 40 μL of sodium acetate (1 N) in a reaction vial (pH 4–4.5). The radiolabeling reaction was carried out under 90 °C for 15 min. The reaction mixture was then diluted with 5 mL of distilled water and passed through a C-18 cartridge (Waters, United States). Trapped on the cartridge, the radioactivity was first washed by 20 mL of distilled water and 20 mL of air. The radioactivity was then eluted into a product vial by 500 μL of ethanol, and 4.5 mL of water was added into the product vial for final formulation. The radiochemical purity of ^68^Ga-**1** was determined on an Agilent 1100 system (Agilent, United States) equipped with a FC3200 gamma detector (Bioscan, United States). A Phenomenex C-18 Luna column was used for HPLC elution with MeCN (Phase A) and 0.1% TFA in water (Phase B) based on a gradient method set as follows (flowrate: 1 mL/min): 0–9 min, 30% A to 70% A; 9–10 min, 70% A to 30% A.

### *In vitro* physicochemical property tests

The stability profile of ^68^Ga-**1** was evaluated in 80% EtOH solution, PBS saline and rat serum as described in previously published literature (Chen et al., 2023). Briefly, about 600 μL of freshly prepared product solutions were added into 2 mL of these test solutions with each 100 μL. These test solutions were kept in a water bath (37 °C) for 4 h, and the samples for stability test were obtained from these solutions at 1, 2, 3 and 4-h after water bath incubation. Samples were loaded into the HPLC system and eluted with the mobile phase as described in the radiolabeling section previously. The protein was denatured by acetonitrile and removed by centrifugation before loading to HPLC for samples from rat serum.

Lipophilicity of a radiotracer is crucial for the biodistribution and Pharmacokinetics properties. The lipophilicity of the radiolabeled compound **1** (^68^Ga-**1**) was determined according to previously published literature and presented as the negative logarithms of the partition coefficient using PBS saline with pH 7.4 (*Log D*_*7.4*_) (Li et al., 2019). Briefly, radioactivity concentrations in PBS saline layer and 1-octanol layer were obtained from a mixture of radiotracer mixture of PBS saline and 1-octanol and were used to calculate the partition coefficient by the ratios of the two concentrations. A series of radiotracer solutions were produced, and the corresponding partition coefficient was tested until a constant value of *log D*_*7.4*_ was reached.

### Cell uptake experiments

H1975^L858R/T790M^, H3255^L858R^ and A549^WT^ cells were purchased from American Type Culture Collection and cultured in the correspond medium recommended by American Type Culture Collection (ATCC). Plasmid-transfected A549 cells that express EGFR^T790M^ mutation were obtained from Haixing Biosciences (Suzhou, China). In cellular uptake studies, cells were cultured and seeded into 60-mm culture dishes with a density of 5 * 10^5^ cells per well overnight for attachment before use. Freshly prepared ^68^Ga-**1** (∼370 KBq, ∼50–100 uL) was added into the medium of cells and incubated at 37 °C for testing. At designated timepoints, i.e., 15-, 30-, 45-, 60-min, the medium was collected, and the well was washed with cold PBS (2 mL). The PBS and medium were combined and tested in the wizard (2470, Perkin Elmer, United States) for radioactivity counts. Cells in the well were treated with trypsin and then collected, counted in the wizard. In cell blocking studies, Rociletinib was added into the well (final concentration of 1 μM was reached in the well) 1 h before the addition of radiolabeled ^68^Ga-**1**, and the subsequent operation procedure was kept identical as described above. Tracer uptake ratios in the cell can be calculated by the following formula: Uptake radios = (radio-counts for the cells)/(radio-counts for the cells + radio-counts for the medium and PBS) * 100%.

### Biodistribution studies

To further evaluate the *in vivo* pharmacokinetics profile of ^68^Ga-**1**, a tissue biodistribution assessment was conducted in normal Kunming mice. All protocols and procedures in the biodistribution study were reviewed and approved by the animal care and use committee of Sichuan University. Briefly, all animals were administered with 30–40 μCi of ^68^Ga-**1** by tail vein injection under isoflurane-induced anesthesia and grouped by timepoints, i.e., 5, 15, 30, 60, 90 and 120 min (n = 5). A gentle stream of air (0.8 L/min) containing 2% of isoflurane gas were generated by a compact anesthesia module (MINERVE, France) and fed into the anesthesia chamber. At designated timepoints, animals were sacrificed by carbon dioxide inhalation, and the organs and tissues of interest were harvested, weighted, and tested in the gamma-counter (2470, Perkin Elmer, United States) for radio-counts. The tissue biodistribution profile of ^68^Ga-**1** can be calculated with the decay-corrected injected dose and presented as the percentage of the injected dose per gram of tissue (%ID/g).

### Micro-PET/CT imaging studies

Preparation of animal models: tumor cells were cultured, harvested and suspended in 100 μL of PBS (approximately 5 * 10^6^ cells). The cells (100 μL suspension) were then injected subcutaneously into the left or right axilla of the BALB/c nude mice (16–20 g, 4–6 weeks). The length of the long axis (a) and short axis of (b) the tumor was measured every 2 days, and the volume of the tumor was estimated by the following formula: Tumor volume = a*b*b/2. Once the volume of the tumor reached 500 mm^3^ (approximately 10–15 days after injection of tumor cells), the PET imaging study was carried out immediately. Briefly, 3.74 MBq of freshly prepared ^68^Ga-**1** was administered into the tumor-bearing mice via tail vein injection with isoflurane-induced anesthesia (details were provided in biodistribution studies section). Static PET imaging was performed at 15-, 30-, 60-, and 120-min post-injection on a micro-PET/CT scanner (IRIS, Inviscan, France), and the data were reconstructed by 3D-OSEM algorithm with a Monte Carlo-base accurate model for attenuation correction. The anesthesia of animals during the scan was maintained by the inhalation of air containing 2% of isoflurane gas (also generated by the compact anesthesia module) by a mice ventilation mask. The regions of interest (ROIs) for organs of interest were manually drawn on the static PET images, and the radio-concentration (KBq/cc) for all ROIs were obtained from software. For blocking studies, subjects were pre-injected with Rociletinib (1 mg/kg) 30 min before the tracer administration.

### Statistical analysis

All data in this study are presented as mean ± standard deviation (Mean ± SD). Statistical analysis was performed with GraphPad Prism 10.1.2 using unpaired independent sample t-tests, and a *P* value <0.05 was considered statistically significant.

## Results

### Molecular docking studies

**FIGURE 2:**
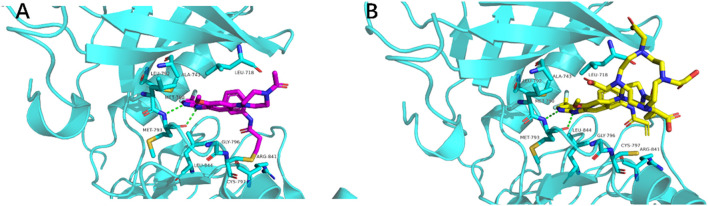
Crystal complex of rociletinib with (PDB: 2JIT) **(A)** and predicated binding of compound **1** with EGFR^T790M^ **(B)**.

Based on the cocrystal structure of EGFR^T790M^ with Rociletinib (PDB: 2JIT), a number of important ligand-protein interactions can be noticed, such as two hydrogen bonds with Met793 carbonyl and amide of the EGFR^T790M^ kinase with the anilinopyrimidine core of Rociletinib, and a hydrophobic interaction of the mutant gatekeeper residue Met790 and the trifluoromethyl substituent on the pyrimidine ring of Rociletinib. In addition, we also noticed the amide that attached to the piperazine ring, which oriented towards the external space of the kinase binding pocket. Therefore, the introduction of a bulky group with significant steric hindrance may not affect the binding potency of compounds with target kinase. In docking studies, compound **1** displayed similar binding mode with Rociletinib with the introduction of DOTA, and overlaid well with Rociletinib, suggesting a comparable high affinity of compound **1** with EGFR^T790M^. In addition, the introduction of DOTA would improve the pharmacokinetics properties of compound **1** compared with Rociletinib as a radiotracer, since DOTA moiety contains multiple hydrophilic centers such as carboxyl groups and nitrogen atoms (Figure 2).

#### Chemical synthesis

Compound **1** was successfully prepared following the previously published literature with minor modifications, as described above in Scheme 1 (Walter et al., 2013; Ward et al., 2013; Fawwaz et al., 2021). Briefly, tert-butyl 4-(4-amino-3-methoxyphenyl)piperazine-1-carboxylate, as a commercially available starting material, reacts with intermediate N-(3-((2-chloro-5-(trifluoromethyl)pyrimidin-4-yl)amino)phenyl)acrylamide for Rociletinib to produce compound **2**. After the deprotection reaction under acidic condition, the unprotected amino group at compound **3** was used for the amide formation reaction with DOTA-GA anhydride to generate the final target molecule (compound **1**, DOTA- Rociletinib). The details for the chemical synthesis procedure and the characterization profile were provided in supporting materials.

### Results for biochemical activity test

Based on the Lantha screen Assay, compound **1** displayed similar enzymatic inhibition activity with Rociletinib. As shown in Table 1, the IC_50_ values for Rociletinib and compound **1** were 8.24 nM and 9.21 nM, respectively. The results indicate that the incorporation of the DOTA as chelating group with larger steric hindrance on the N atom of the piperazinyl group in compound **1** showed nearly no impact on the enzymatic inhibitory activity against EGFR^T790M^. Based on molecular docking studies and biochemical testing, compound 1 was identified as a promising radiolabeled PET tracer targeting EGFR^T790M^ and the subsequent radiolabeling and evaluations were carried out thereafter.

**TABLE 1:** The IC_50_ values for rociletinib and compound 1 against EGFR^T790M^ in lantha screen assay.

Compound	IC_50_ (nM)
Rociletinib	8.24 ± 0.91
**1**	9.21 ± 0.82
Staurosproine	0.39 ± 0.04

#### ^68^Ga radiolabeling

**FIGURE 3:**
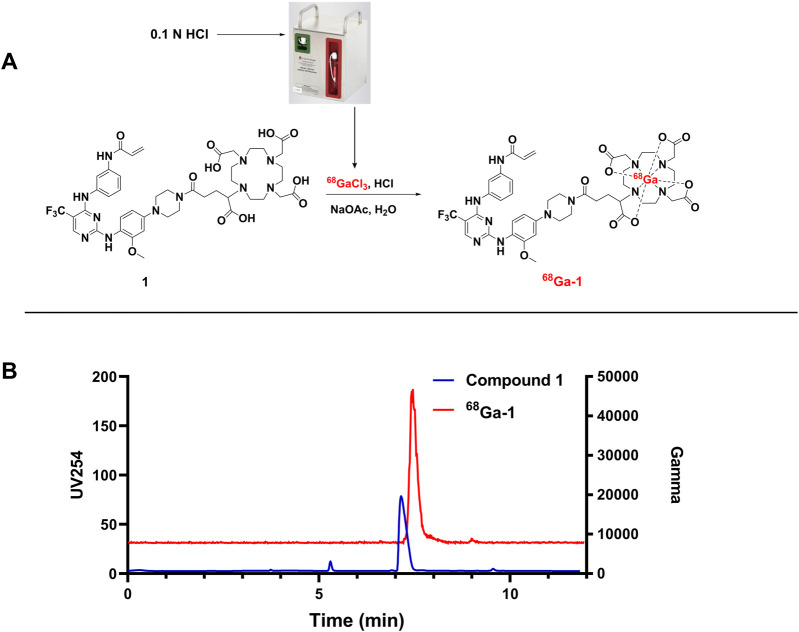
^68^Ga Radiolabeling reaction for compound 1 **(A)** and the co-injection of final product with the precursor **(B)**.

Radiolabeling of Compound **1** was successfully achieved with high radiochemical yield (92.93% ± 2.57%, n = 5) under 90 °C for 10 min. The radiochemical purity of ^68^Ga**-1** was greater than 95% after the C18 cartridge based SPE purification procedure as displayed in Figure 3. The retention time of ^68^Ga-**1** was 7.8 min and the unlabeled precursor of compound **1** is 7.6 min, respectively.

##### *In vitro* physicochemical property tests

Based on the analytical HPLC analysis of samples from ^68^Ga-**1** solutions of 80% EtOH, PBS saline and rat serum, ^68^Ga-**1** displayed high *in vitro* stability. Over 90% intact ^68^Ga-**1** was identified from the HPLC diagram with these test solutions incubated at 37 °C. According to the radio-concentrations for the PBS and 1-octanol, the *log D*_*7.4*_ value for ^68^Ga-**1** was −1.68 ± 0.24, indicating a more hydrophilic property compared to Rociletinib, which displayed a *log P* value of 3.95 from the ChemBioDraw 20.0 predictions.

#### Cell uptake experiments

**FIGURE 4:**
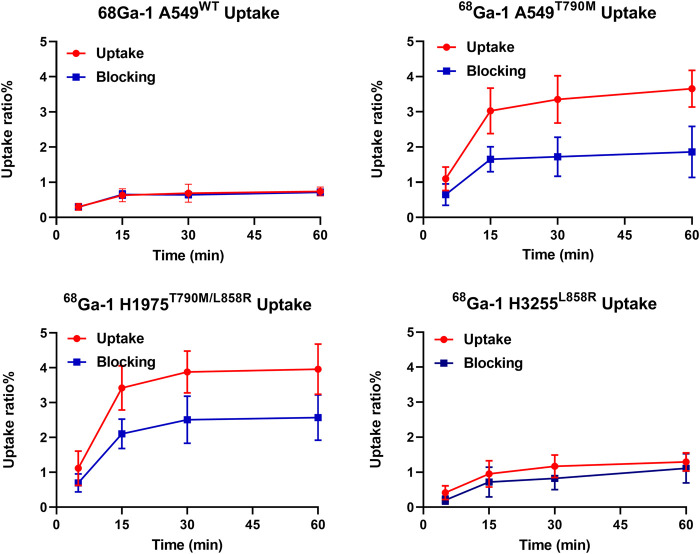
Results for cell uptake experiments of ^68^Ga-**1** (n = 3).

The tracer uptake values for all cells are expressed as the ratios of radio-counts in cells to the sum of radio-counts added into the well, as presented in Figure 4. ^68^Ga-**1** displayed higher accumulation in H1975 cells (bearing EGFR^T790M/L858R^) and A549-EGFR^790M^ (with EGFR^T790M^) compared with H3255 cells (EGFR^L858R^) and A549 (EGFR^WT^). After 2-h incubation, tracer uptake ratio for A549^WT^, H3255^L858R^, H1975^T790M/L858R^ and A549^T790M^ cells were 0.74% ± 0.10%, 1.29% ± 0.21%, 3.96% ± 0.59% and 3.65% ± 0.42% (n = 3), respectively. In addition, with the addition of Rociletinib as blocking agent, a remarkable decrease in tracer uptake ratios was observed in these cells, as also displayed Figure 4. In H1975^T790M/L858R^ and A549^T790M^ cells, tracer uptake ratios decreased to 2.57% ± 0.53% and 1.86% ± 0.59% (n = 3), with blocking efficiencies of 35% and 49% for two cells, respectively. However, only 3% and 14% blocking efficiencies were observed in A549^WT^ and H3255 cells. These results indicated a strong correlation between the EGFR^T790M^ expression and tracer uptake in cell experiments.

### Biodistribution studies

**FIGURE 5:**
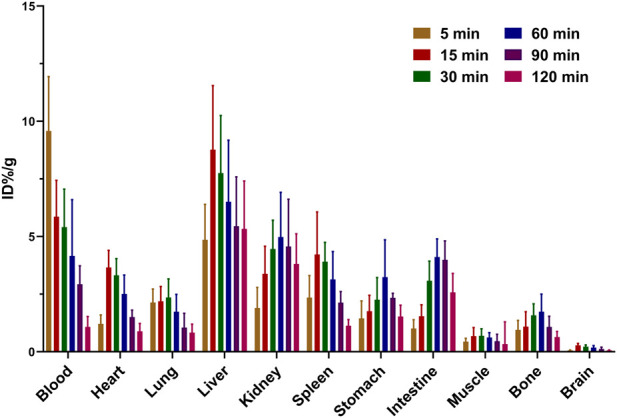
Biodistribution profile of ^68^Ga-**1** in normal Kunming mice (n = 5).

The tissue biodistribution properties of ^68^Ga-**1** in Kunming mice are expressed as %/ID/g in each time point, as displayed in Figure 5 (n = 5, and the detailed quantitative data were provided in SI). ^68^Ga-**1** showed a fast pharmacokinetics profile, and the radioactivity in blood, liver, kidney and other major organs showed a fast clearance during the 120-min experiment. The liver displayed highest radioactivity accumulation at 15 min post injection (p.i.) with 8.77% ± 2.78% ID/g, and the radioactivity accumulation decreased to 5.33% ± 2.08% ID/g at 120 min p.i. In addition, small intestine and spleen displayed a significant radioactivity uptake during the experiment, with peak uptake value of 4.11% ± 0.79% ID/g and 3.14% ± 1.21% ID/g at 60 min p.i., suggesting a major hepatobiliary excretion of this compound in mice. A significant accumulation of radioactivity in kidney was also noticed, with peak uptake value of 4.98% ± 1.94% ID/g at 60 min p.i. and 3.81% ± 1.31% ID/g at 120 min p.i., respectively. Thus, a minor urinary excretion of ^68^Ga-**1** can be inferred according to the accumulation of radioactivity in the kidney. Other major organs, such as the lung, heart, muscle and brain, showed no abnormal radioactivity accumulation, with peak uptake values of 1.74% ± 0.75%, 2.51% ± 0.82%, 0.62% ± 0.21% and 0.18% ± 0.09% ID/g at 60 min p.i., respectively. A small amount accumulation of radioactivity in the bone was observed, indicating a minor decomposition of ^68^Ga-1 *in vivo*. With the biodistribution profile of ^68^Ga-**1** in hand, the subsequent PET imaging study was performed thereafter.

#### Micro-PET/CT imaging studies

**FIGURE 6:**
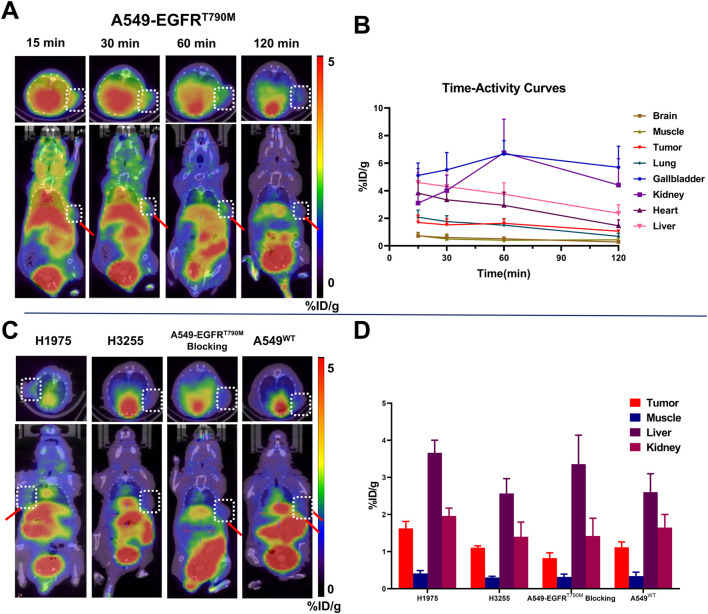
Micro-PET studies for ^68^Ga-**1** in mice models (n = 3). **(A)** Static PET images of A549-EGFR^T790M^ mice models at different time points; **(B)** Time-activity curves generated from A549-EGFR^T790M^ mice PET images; **(C)** Representative static PET images for H1975, H3255, A549^WT^ and A549-EGFR^T790M^ (blocked with Rociletinib) mice models at 60 min post-injection; **(D)** Selected organ uptake value for different tumor models at 60 min post-injection. Red arrows denote the tumor.

The representative PET images for ^68^Ga-**1** in different tumor models and the corresponding Time-activity curves are presented in Figure 6 (n = 3). Radioactivity accumulation in tumor mice well agreed with the biodistribution profile, with significant initial accumulation of radio-signal in the liver, kidney, lower digestive tract, spleen and bladder observed in all kinds of tumor mouse models investigated in this study. The bladder, gallbladder, kidney and liver displayed the highest tracer accumulation, indicating a major urinary excretion and minor hepatobiliary metabolism of ^68^Ga-**1**. For A549-EGFR^T790M^ and H1975^L858R/T790M^ mice models, tumor regions were clearly visualized. However, in H3255^L858R^ and A549^WT^ mice models, lower radioactivity accumulation in tumors were observed as well as mice from blocking studies (A549-EGFR^T790M^ mice models blocked with Rociletinib).

As displayed in Figures 6A,B, an initial high uptake in gallbladder, kidney and liver were observed in A549-EGFRT^790M^ mice models with %ID/g value of 5.11 ± 0.73, 3.10 ± 0.59 and 4.60 ± 0.58, respectively. A fast uptake of radiotracer in tumor can be noticed and maintained afterwards, with %ID/g values of 1.62 ± 0.38 at 15 min p.i. and 1.56 ± 0.16 at 60 min p.i., respectively. With the gradually clearance of radio-signal from major organs and background such as lung and muscle, the “Tumor-to-muscle (T/M)” ratio peaked at 60 min p.i. with highest value of 3.82 during the entire scan. The tumor uptake displayed a decrease after 60 min p.i., with %ID/g value of 1.06 ± 0.10 and T/M ratio of 2.46 obtained at 120 min p.i., respectively. However, the gallbladder exhibited highest uptake (peaked at 60 min p.i. with 6.67% ± 0.78% ID/g) which may due to the lipophilicity of the pyrimidine scaffold at all time points. Based on the PET images and *in vivo* distribution profile of ^68^Ga-**1** obtained in A549-EGFR^T790M^ mice models, the subsequent PET image studies were performed at 60 min p.i. for H1975, H3255 and A549^WT^ mice models and in blocking studies.

In EGFR^T790M^ positive H1975 mice models, a similar tumor uptake was observed with %ID/g value of 1.65 ± 0.13 at 60 min p.i., as well as a T/M ratio of 3.96. The other major organs also displayed similar tracer uptake profiles, as displayed in Figures 6C,D. In blocking studies with A549-EGFR^T790M^ mice models, the pre-injection of Rociletinib significantly inhibited the tumor uptake of ^68^Ga-**1** (P < 0.05), with the uptake value decreasing to 0.83 ± 0.12 at 60 min p.i., while other major organs displayed comparable uptake values compared with non-blocking subjects. In addition, ^68^Ga-**1** also exhibited a lower tumor uptake in H3255 mice models with EGFR^L858R^ mutations and A549 mice models with EGFR^WT^. As also displayed in Figures 6C,D, an average uptake value of 1.10% ± 0.04% ID/g (H3255 mice models) and 1.12% ± 0.12% ID/g (A549 mice models) was respectively observed at 60 min p.i., compared with that of A549-EGFR^T790M^ mice models (1.62% ± 0.38% ID/g) and H1975 mice models (1.65% ± 0.13% ID/g). Together with the uptake profile obtained in blocking studies, the results in A549-EGFR^T790M^ mice models indicated the tumor uptake of ^68^Ga-**1** is selective. In all subjects investigated in these studies, no abnormal uptake of radio-signal in other major organs was observed, indicating the *in vivo* stability of ^68^Ga-**1**.

## Discussion

As a subfamily member of receptor tyrosine kinase, EGFR was regarded as the most important target for anti-tumor therapies for NSCLC patients in last decades. The genotyping of EGFR is also of great importance for personalizing NSCLC therapy and predicting therapy efficacy. Although over 30 radiotracers have been reported to possess clinical potential to visualize EGFR mutations (such as ex20ins, 19-Del and 21-L858R) *in vivo*, few PET tracers have been reported with the ability to detect EGFR^T790M^ mutation. Developing highly sensitive and selective EGFR^T790M^ PET tracers for the complement of current EGFR targeted PET tracers is of great importance for scientific research and clinical applications. Bearing the pyrimidine scaffold, Rociletinib held potent affinity against EGFR^T790M^, and our interest in this compound lies in the convenient modification and conversion of Rociletinib, to obtain the target molecule as a^68^Ga-labeled PET tracer using DOTA moiety as a chelator group. ^68^Ga-**1** was produced with high radiochemical yield, and the *in vitro* evaluations indicated it possessed strong stability and suitable lipophilicity, which is important for further *in vivo* evaluations. The selective binding of ^68^Ga-**1** with EGFR^790M^ was confirmed by cell uptake experiments with EGFR^T790M^ positive and wild type cells, as well as blocking studies. In H1975 (EGFRT^790M/L858R^) and A549-EGFR^T790M^ cells, tracer uptake is twofold higher than that of EGFR^WT^ cells, and the uptake can be effectively blocked by Rociletinib. Compared with maximum cell uptake of 6% with ^18^F-FEA-Erlotinib (Huang et al., 2018) and 5% of ^18^F-MPG (Sun et al., 2018), 4% of ^68^Ga-**1** seems reasonable for this type of TKI tracer. The relatively low absolute percentage of cell uptake may be associated with cell membrane permeability and membrane protein expression (Kell, 2021).

Biodistribution studies revealed the hepatobiliary/urinary excretion of ^68^Ga-**1** *in vivo*, as well as a moderate pharmacokinetics property. In micro-PET/CT studies, ^68^Ga-**1** also displayed selective accumulation in EGFRT790M positive tumors with highest uptake observed (1.62% ± 0.38% ID/g in A549-EGFR^T790M^ tumors and 1.65% ± 0.13% ID/g in H1975 tumors) at 60 min p.i., and an effective blocking efficacy (49.7%) was observed in blocking studies. However, tracer accumulation was also observed in A549 EGFR^WT^ tumor models (1.12% ± 0.12% ID/g) and H3255 EGFR^L858R^ tumor models (1.10% ± 0.04% ID/g), compared with 1.62%–1.63% ID/g in EGFR^T790M^ positive tumors, making it challenging to identify EGFR^T790M^ from other EGFR mutations or wild type EGFR. In addition, a relatively higher background uptake in bladder, gallbladder, liver, kidney and lung was noticed in both biodistribution and micro-PET/CT imaging studies, suggesting a higher plasma protein binding, nonspecific binding and lack of target engagement of ^68^Ga-**1**.

Suitable hydrophilicity of a PET tracer is essential for the *in vivo* pharmacokinetics properties, and most current successful 68Ga labeled PET tracer held strong hydrophilicity, such as ^68^Ga-PSMA-11 (log *P:* −2.91 ± 0.0631) and ^68^Ga-FAPI-46 (log *D*_*7.4*_: −3.38 ± 0.01) (Baranski et al., 2018; Kogler et al., 2025). Adequate hydrophilicity of a compound would lead to fast background clearance via kidney and potential membrane penetration, which is important for target engagement. Insufficient hydrophilicity of a compound usually related to high plasma protein binding and nonspecific binding, which would lead to higher and longer retention of background uptake in liver, kidney, gallbladder and lower gastrointestinal tract. Therefore, according to the log *P* value of ^68^Ga-**1** and the PET images obtained in this study, we conclude that the insufficient hydrophilicity of compound **1** impairs both background clearance and the membrane penetration necessary for target engagement. In addition, although compound **1** and Rociletinib displayed potent *in vitro* enzymatic activity against EGFR^T790M^, Rociletinib exhibited limited specificity against EGFR^WT^ with a *Ki* value of 303.3 nM in a previously reported literature (compared with that of 21.5 nM against EGFR^T790M/L858R^ in same study) (Walter et al., 2013). Thus, as an analogue of Rociletinib, the *in vivo* specificity of ^68^Ga-**1** would be comprised by the insufficient selectivity against EGFR^WT^ and other alterations of EGFR.

Therefore, further structural modification and optimization of this compound should be focused on the improvement of binding potency on EGFR^T790M^ and selectivity against EGFR^WT^ and other EGFR alterations, and the following options will be beneficial: 1. extensive structural-activity-relationship studies for screening of ideal compounds; 2. chelator/linker optimization to improve affinity; 3. other strategies to enhance the tumor retention and “Tumor to background” ratios such as covalent targeted radioligand technology (Cui et al., 2024). In addition, the optimization of physicochemical properties by enhancement of hydrophilicity would also be of great importance for this tracer to improve the target accumulation and background clearance. Moreover, with DOTA moiety as a chelator to enable radiometal labeling in compound **1**, further therapeutic radionuclides may also be introduced to the “Aniline-Pyrimidine-Aniline” scaffold to form theragnostic radiopharmaceuticals.

## Conclusion

In conclusion, ^68^Ga-**1** was successfully synthesized and demonstrated promising preliminary characteristics; however, its specificity toward EGFR^T790M^ mutations requires further investigation and validation. ^68^Ga-**1** displayed *in vitro* and *in vivo* specificity toward EGFR^T790M^ compared with EGFR^WT^ and EGFR^L858R^. However, the insufficient selectivity against EGFR^WT^ and other EGFR alterations of ^68^Ga-**1** making it challenging to identify EGFR^T790M^ *in vivo*. Therefore, further structural modification and optimization, focused on the selectivity and physicochemical parameters of this compound, would be needed to develop more successful EGFR^T790M^ selective targeted PET tracer for NSCLC diagnosis and treatment.

## Data Availability

The original contributions presented in the study are included in the article/supplementary material, further inquiries can be directed to the corresponding author/s.
