# Supplementary material for: Synthesis, radiolabeling, and evaluation of a 68Ga-labeled tyrosine kinase inhibitor for detecting EGFRT790M mutations in vivo
Source: Front Bioeng Biotechnol. 2026 Jan 21;14:1741512. doi: 10.3389/fbioe.2026.1741512 (PMC12868298; doi:10.3389/fbioe.2026.1741512)
Supplement: Supplementary file 1 [file DataSheet1.docx]

**Supplementary Materials**

**Synthesis, radiolabeling, and evaluation of a ^68^Ga-labeled Tyrosine Kinase Inhibitor for detecting EGFR^T790M^ Mutations in vivo**

Jiajun Xie^1, 2#^, Weiguo Xu^3#^, Hui Deng^2^, Xiaoai Wu^4, 5*^, Jing Zhu^1, 2, 3*^

1. NHC Key Laboratory of Nuclear Technology Medical Transformation (MIANYANG CENTRAL HOSPITAL)
2. Precision Medicine Key Laboratory of Sichuan Province, West China Hospital of Sichuan University, Chengdu, China, 610041
3. Department of Respiratory and Critical Care Medicine, Mianyang Central Hospital, School of Medicine, University of Electronic Science and Technology of China, Mianyang, China
4. Department of Nuclear Medicine, West China Hospital, Sichuan University, Chengdu, China
5. Sichuan Provincial Engineering Research Center of Radiopharmaceutical Clinical Translation, Sichuan University, Chengdu, Sichuan, 610041, China

Contents

[1. Synthesis of compound 2 3](#_Toc216725483)

[2. Synthesis of compound 3 3](#_Toc216725484)

[3. Synthesis of compound 1 4](#_Toc216725485)

[4. Quantitative mice biodistribution data: 7](#_Toc216725486)

1. **Synthesis of compound 2**

*Tert-butyl4-(4-((4-((3-acrylamidophenyl)amino)-5-(trifluoromethyl)pyrimidin-2-yl)amino)-3-methoxyphenyl)piperazine-1-carboxylate (2):* N-(3-((2-chloro-5 -(trifluoromethyl)pyrimidin -4-yl)amino)phenyl)acrylamide (100 mg, 0.29 mmol, 1.0 eq), tert-butyl 4-(4-amino-3-methoxyphenyl)piperazine-1-carboxylate (117 mg, 0.38 mmol, 1.3 eq) and trifluoroacetic acid (27 μL, 0.35 mmol, 1.2 eq**)** were dissolved in 1 mL of 2-butanol. The reaction mixture was then heated to 100 ^o^C for overnight. Cooling to room temperature, the reaction mixture was extracted by 2 mL of water and ethyl acetate (2 mL *3). The organic layer was combined and dried by anhydrous sodium sulfate, and the solvents were removed by rotary evaporation under reduced pressure. The crude product was then purified by silica gel column chromatography (dichloromethane: methanol= 20 :1) to obtain the compound **2** as a black solid (55 mg, yield 30.7%).

1. **Synthesis of compound 3**

*N-(3-((2-((2-methoxy-4-(piperazin-1-yl)phenyl)amino)-5-(trifluoromethyl)pyrimidin-4-yl)amino)phenyl)acrylamide (3):* To a mixture of compound **2** (55 mg, 0.09 mmol, 1.0 eq) and 2 mL of dichloromethane was added 31 μL (0.4 5mmol，5.0 eq) of trifluoroacetic acid. The reaction mixture was kept stirring at room temperature for 3 hours. The solvent was then removed under reduced pressure, and the crude product was purified by silica gel column chromatography (dichloromethane: methanol = 10 :1) to obtain the compound **3** as yellow solid (39 mg, yield 84.8%).

^1^H NMR (400 MHz, Methanol-d4) δ 8.25 (s, 1H), 7.77 – 7.59 (m, 3H), 7.36 (td, J = 8.1, 1.6 Hz, 1H), 7.14 (d, J = 7.9 Hz, 1H), 6.62 (d, J = 2.6 Hz, 1H), 6.48 – 6.16 (m, 3H), 5.78 (dt, J = 9.5, 2.0 Hz, 1H), 3.84 (d, J = 1.6 Hz, 3H), 3.40 – 3.30 (m, 9H).


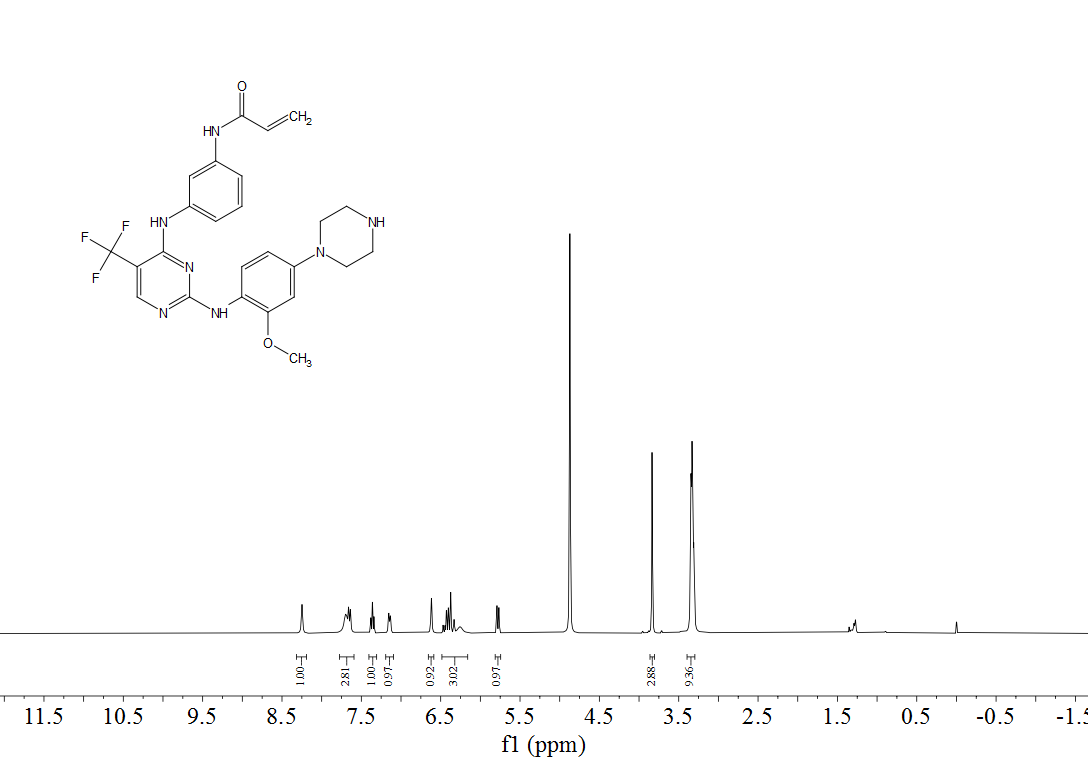


1. **Synthesis of compound 1**

*2,2',2''-(10-(4-(4-(4-((4-((3-acrylamidophenyl)amino)-5-(trifluoromethyl)pyrimidin-2-yl)amino)-3-methoxyphenyl)piperazin-1-yl)-1-carboxy-4-oxobutyl)-1,4,7,10-tetraazacyclododecane-1,4,7-triyl)triacetic acid (1):* To a mixture of compound 2 (39 mg, 0.07 mmol, 1.0 eq), DOTA-GA anhydrous (42 mg, 0.09 mmol, 1.2 eq) and 0.5 mL of DMSO was added ET_3_N (12 μL, 0.08 mmol, 1.1 eq). The reaction mixture was kept stirring at room temperature overnight. Semi-preparative HPLC was used for the purification of the final product with a PhenomenexC18 column (Luna 10u C18, 100A, 250 * 10 mm) on an Alltech 426 pump system. The column was eluted with 10% of MeCN and 90% of water (0.1% HCOOH) at a flow rate of 4 mL/min. The retention time of the final product was 9-10 minutes, and the solutions of the final product were converted into a white powder by lyophilization (26 mg, yield 53.1%). MS calculated for [M+H]+ 972.4, found 971.6; [M+K]+ 1010.3, found 1009.6.

^1^H NMR (400 MHz, DMSO-d6) δ 8.23 (d, J = 35.7 Hz, 2H), 7.51 (dd, J = 21.7, 14.1 Hz, 2H), 7.29 – 7.07 (m, 2H), 6.58 (d, J = 15.5 Hz, 2H), 6.25 (d, J = 17.0 Hz, 2H), 5.74 (d, J = 10.1 Hz, 1H), 3.77 (s, 3H), 3.59 – 2.60 (m, 35H).


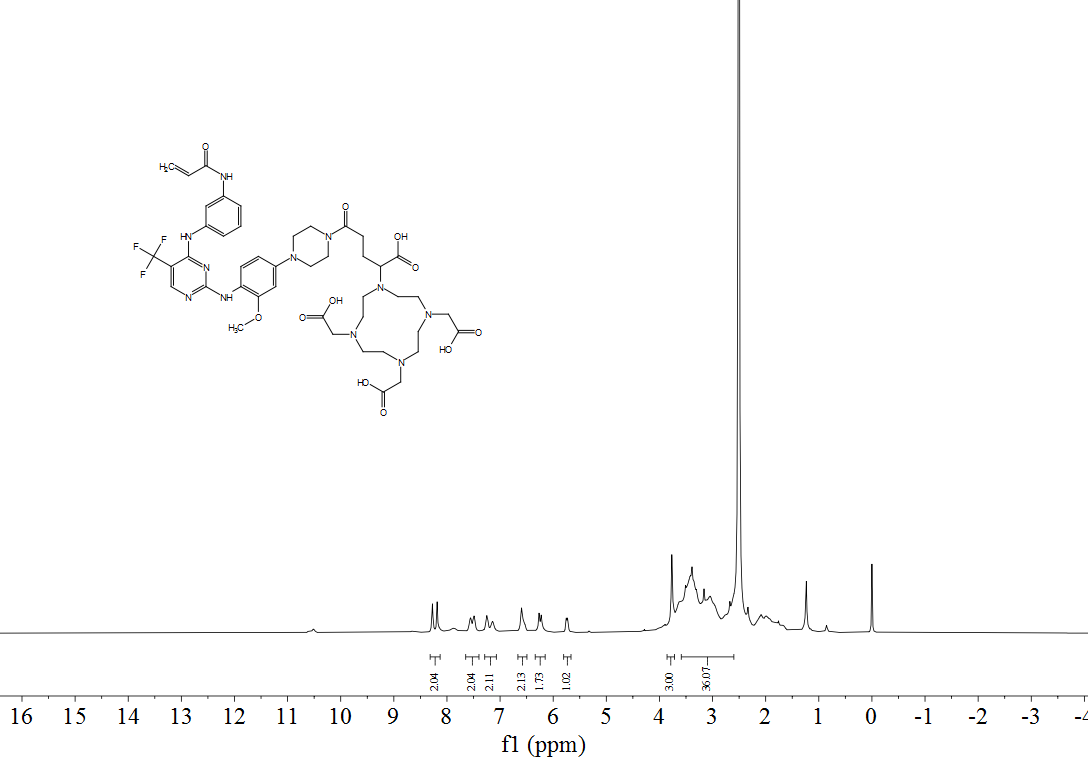


^13^C NMR (101 MHz, DMSO-d6) δ 163.66, 163.66, 163.66, 163.66, 163.56, 139.51, 132.52, 128.89, 128.89, 128.81, 127.89, 127.15,126.34, 123.96, 116.24, 107.50, 107.50, 101.02, 101.02, 72.90, 72.90, 63.42, 63.42, 63.42, 56.10, 56.10, 56.10, 53.01, 53.01, 49.89, 49.47, 44.72, 36.09, 32.34, 29.64, 29.14, 22.49.


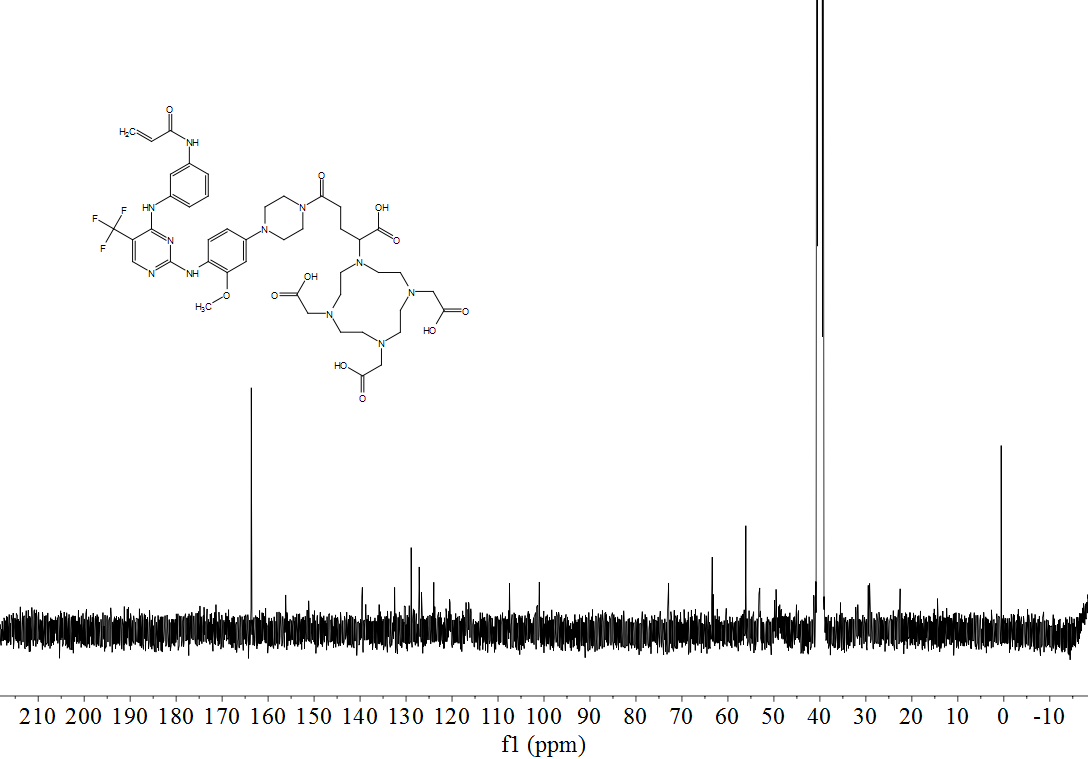


1. Quantitative mice biodistribution data (n = 5):

|  | 5 min | | 15 min | | 30 min | | 60 min | | 90 min | | 120 min | |
| --- | --- | --- | --- | --- | --- | --- | --- | --- | --- | --- | --- | --- |
|  | Mean | SD | Mean | SD | Mean | SD | Mean | SD | Mean | SD | Mean | SD |
| Blood | 9.58 | 2.36 | 5.86 | 1.58 | 5.41 | 1.65 | 4.16 | 2.44 | 2.93 | 0.8 | 1.08 | 0.45 |
| Heart | 1.21 | 0.39 | 3.66 | 0.74 | 3.32 | 0.72 | 2.51 | 0.82 | 1.51 | 0.3 | 0.89 | 0.34 |
| Lung | 2.14 | 0.58 | 2.19 | 0.64 | 2.36 | 0.8 | 1.74 | 0.75 | 1.05 | 0.62 | 0.83 | 0.37 |
| Liver | 4.86 | 1.54 | 8.77 | 2.78 | 7.75 | 2.5 | 6.51 | 2.67 | 5.45 | 2.14 | 5.33 | 2.08 |
| Kidney | 1.9 | 0.89 | 3.38 | 1.2 | 4.46 | 1.25 | 4.98 | 1.94 | 4.57 | 2.05 | 3.81 | 1.31 |
| Spleen | 2.35 | 0.96 | 4.22 | 1.85 | 3.91 | 0.84 | 3.14 | 1.21 | 2.13 | 0.48 | 1.13 | 0.27 |
| Stomach | 1.45 | 0.76 | 1.76 | 0.69 | 2.26 | 0.96 | 3.24 | 1.62 | 2.34 | 0.2 | 1.53 | 0.49 |
| Intestine | 1.01 | 0.38 | 1.54 | 0.5 | 3.08 | 0.85 | 4.11 | 0.79 | 3.99 | 0.82 | 2.58 | 0.82 |
| Muscle | 0.44 | 0.15 | 0.68 | 0.37 | 0.69 | 0.31 | 0.62 | 0.21 | 0.46 | 0.3 | 0.33 | 0.97 |
| Bone | 0.95 | 0.41 | 1.09 | 0.65 | 1.58 | 0.5 | 1.74 | 0.76 | 1.08 | 0.46 | 0.64 | 0.24 |
| Brain | 0.06 | 0.02 | 0.28 | 0.08 | 0.22 | 0.08 | 0.18 | 0.09 | 0.12 | 0.08 | 0.06 | 0.02 |
